# Augmented renal clearance is not a risk factor for mortality in *Enterobacteriaceae* bloodstream infections treated with appropriate empiric antimicrobials

**DOI:** 10.1371/journal.pone.0180247

**Published:** 2017-07-05

**Authors:** Jason P. Burnham, Scott T. Micek, Marin H. Kollef

**Affiliations:** 1 Division of Infectious Diseases, Washington University School of Medicine, St. Louis, Missouri, United States of America; 2 St. Louis College of Pharmacy, St. Louis, Missouri, United States of America; 3 Division of Pulmonary and Critical Care Medicine, Washington University School of Medicine, St. Louis, Missouri, United States of America; University of Florida, UNITED STATES

## Abstract

The main objective of the study was to assess whether augmented renal clearance was a risk factor for mortality in a cohort of patients with *Enterobacteriaceae* sepsis, severe sepsis, or septic shock that all received appropriate antimicrobial therapy within 12 hours. Using a retrospective cohort from Barnes-Jewish Hospital, a 1,250-bed teaching hospital, we collected data on **i**ndividuals with *Enterobacteriaceae* sepsis, severe sepsis, and septic shock who received appropriate initial antimicrobial therapy between June 2009 and December 2013. Clinical outcomes were compared according to renal clearance, as assessed by Modification of Diet in Renal Disease (MDRD) and Chronic Kidney Disease Epidemiology Collaboration (CKD-EPI) formulas, sepsis classification, demographics, severity of illness, and comorbidities. We identified 510 patients with *Enterobacteriaceae* bacteremia and sepsis, severe sepsis, or septic shock. Sixty-seven patients (13.1%) were nonsurvivors. Augmented renal clearance was uncommon (5.1% of patients by MDRD and 3.0% by CKD-EPI) and was not associated with increased mortality. Our results are limited by the absence of prospective determination of augmented renal clearance. However, in this small cohort, augmented renal clearance as assessed by MDRD and CKD-EPI does not seem to be a risk factor for mortality in patients with *Enterobacteriaceae* sepsis. Future studies should assess this finding prospectively.

## Introduction

Augmented renal clearance (ARC) is the term given to the phenomenon of accelerated glomerular filtration resulting in reduced systemic exposure to renally eliminated drugs. The incidence of ARC varies by population studied, being more prevalent in persons with traumatic injuries, persons of younger age, males, and sepsis patients with lower acute physiology and chronic health evaluation (APACHE) II scores [1–4]. While there is no universal agreement on how to best measure ARC [5–9], its effect on antibiotic levels is now well established [10–13]. Multiple studies have shown that patients with ARC have reduced exposure to renally cleared antibiotics, but only one study has shown any effect on outcome [1, 10, 14]. We previously described a cohort of patients with *Enterobacteriaceae* sepsis, severe sepsis, or septic shock that all received appropriate antibiotics within 12 hours of positive blood cultures and found that cirrhosis, African-American race, and presence of septic shock were risk factors for mortality [15]. As these three factors have been associated with hyperdynamic cardiac output and thereby potentially ARC, we performed a secondary analysis of our previous cohort with the goal of determining whether ARC was prevalent and whether it impacted mortality in a group of patients all receiving appropriate empiric antibiotic therapy.

## Materials and methods

### Study location and patient population

This study was conducted at Barnes-Jewish Hospital, a 1250 bed academic medical center located in St. Louis, MO. This was a secondary analysis of a cohort that we previously described [15]. The study period was June 1, 2009 through December 31, 2013, corresponding to the length of time for which an electronic medical record was available that could verify time of antibiotic administration. All consecutive hospitalized patients with sepsis, severe sepsis, or septic shock and a positive blood culture for an organism in the *Enterobacteriaceae* family during the study period were analyzed for eligibility. This study was approved by the Washington University School of Medicine Human Studies Committee.

### Study design and data collection

The cohort and data collection has been previously described [15]. This was a retrospective cohort study of all patients age ≥ 18 with sepsis, severe sepsis, or septic shock (as defined by systemic inflammatory response syndrome (SIRS) criteria) and a positive blood culture for an organism in the *Enterobacteriaceae* family. Patients were included only if they had positive blood cultures with a single organism from the *Enterobacteriaceae* family. ICD-9 codes indicative of acute organ dysfunction or the need for vasopressors were used to classify patients as having severe sepsis or septic shock, respectively. The primary endpoint was all-cause 30-day mortality, calculated from the time that a positive blood culture was drawn. Only the first episode of sepsis, severe sepsis, or septic shock was evaluated. Baseline characteristics, including age, gender, race, place of origin, healthcare exposure, receipt of antibiotics within 30 days of positive culture, presence of immunosuppression, Acute Physiology and Chronic Health Evaluation (APACHE) II [16] scores (calculated based on clinical data present during the 24 hours after positive blood cultures were drawn), Charlson Comorbidity Index, and medical comorbidities were obtained.

### Definitions

For augmented renal clearance analysis, the highest creatinine in the 24 hours before or after the time at which a positive blood culture was drawn was collected. Augmented renal clearance was defined as an estimated glomerular filtration rate (GFR) of >130 mL/min/1.73 m^2^. All GFRs were calculated using both the Modification of Diet in Renal Disease (MDRD) and Chronic Kidney Disease Epidemiology Collaboration (CKD-EPI) formulas [17, 18]. We also performed a sensitivity analysis with a GFR cutoff of 100 mL/min/1.73 m^2^ given prior reports of underestimation of true GFR by the MDRD and CKD-EPI equations [5].

All patients received appropriate initial antibiotic therapy, defined as antibiotics that had *in vitro* activity against the cultured organism (and were not single-agent aminoglycosides), that was administered within 12 hours of when a positive blood culture was drawn and continued for at least 24 hours. Appropriateness of therapy after the initial 24 hours was not assessed. For extended-spectrum β-lactamase producing organisms, initial use of a carbapenem was required to be classified as appropriate treatment. Antimicrobial susceptibilities were determined using disc diffusion methodology. Appropriate antibiotics administered ≤ 12 hours before positive blood cultures were drawn were considered to have a time of administration of 0 minutes.

Only the first episode of bacteremia during a hospitalization was considered. Patients who had an episode of bacteremia during their hospitalization prior to *Enterobacteriaceae* bacteremia were excluded (only two cases, one with *Staphylococcus epidermidis*, one with *Enterococcus*). The following organisms were considered contaminants if not recultured within 72 hours: coagulase-negative *Staphylococci*, *Corynebacterium*, *Propionibacterium acnes*, or Viridans group *Streptococcus*. Patients were excluded if they were under 18 years of age or if they had a blood culture positive for more than one organism. All patients who did not receive antibiotics within 12 hours of when positive blood cultures were drawn were excluded. Discharge on hospice was considered a mortality equivalent. All patients discharged on hospice were considered to expire at the time of hospital discharge.

### Antimicrobial monitoring

From January 2002 through the present, BJH has utilized an antibiotic control program to help guide antimicrobial therapy for bacterial infections. During this time, the use of cefepime, gentamicin, or vancomycin was unrestricted. However, initiation of intravenous ciprofloxacin, imipenem, meropenem, piperacillin/tazobactam, ceftolozane/tazobactam, ceftazidime/avibactam, linezolid, ceftaroline, or daptomycin was restricted and required preauthorization from either a clinical pharmacist or infectious diseases physician. Each intensive care unit (ICU) and non-ICU unit also had a clinical pharmacist who reviewed all antibiotic orders to insure that dosing and interval of antibiotic administration was adequate for individual patients based on body size, renal function, and the resuscitation status of the patient.

The initial antibiotic dosages employed for the treatment of bacterial infections at BJH are as follows: cefepime, 1 to 2 g every 8 hours; piperacillin–tazobactam, 4.5 g every 6 hours; imipenem, 0.5 g every 6 hours; meropenem, 1 to 2 g every 8 hours; ceftolozane/tazobactam, 1.5 or 3 g every eight hours; ceftazidime/avibactam, 2.5 g every 8 hours; ciprofloxacin, 400 mg every 8 hours; levofloxacin, 750 mg once daily; vancomycin, 15 mg/kg every 12 hours; linezolid, 600 mg every 12 hours; and ceftaroline, 600 mg every 8 hours.

### Antimicrobial susceptibility testing

The microbiology laboratory performed antimicrobial susceptibility of the bacterial isolates using the disk diffusion method according to guidelines and breakpoints established by the Clinical Laboratory and Standards Institute (CLSI) and published during the inclusive years of the study [19]. All classifications of antibiotic resistance were based on *in vitro* susceptibility testing using these established breakpoints. Our laboratory routinely and expeditiously adopts updates to CLSI breakpoints. All breakpoints used for analysis in this study were those recommended by the CLSI at the time cultures were drawn.

### Statistical analysis

Univariate analysis was performed by chi-square or Fischer’s exact test where appropriate for categorical values. Student’s t-test or Mann-Whitney U test was used where appropriate for continuous variables. Continuous variables were reported as means with standard deviations or median with interquartile range for non-normally distributed variables. Categorical data were expressed as frequencies. A p-value of <0.05 was considered significant. Using our previous multivariate logistic regression model [15], we attempted to force the presence of augmented renal clearance into the model to determine if it changed the model. To determine risk factors for ARC, we performed binomial logistic regression using forward selection methodology using variables with p value of <0.10. Variables were assessed for collinearity. Goodness-of-fit was assessed via the Hosmer-Lemeshow c-statistic. All tests were two-tailed. We also performed a sensitivity analysis in which patients who received ceftriaxone were excluded, as its clearance should not be affected significantly by renal clearance. All analysis was done using SPSS v24 (IBM, Armonk, NY).

## Results

Five-hundred ten patients with sepsis, severe sepsis, or septic shock (by SIRS criteria) due to *Enterobacteriaceae* met the inclusion criteria. After exclusion of 16 patients with end-stage renal disease, there were 494 patients for which GFR was calculated. Baseline characteristics of the patients stratified by GFR are listed in Table 1. Augmented renal clearance was present in a minority of patients using values obtained from MDRD (5.1%, n = 25) and CKD-EPI (3.0%, n = 15) calculations. In univariate analysis, age, APACHE II and Charlson Comorbidity scores, and presence of CHF or African-American race were significantly different between the ARC and no ARC groups. Age, Charlson comorbidity and APACHE II scores were lower in patients with ARC. African-American race was more common in the ARC group, whereas CHF was absent. In our sensitivity analysis, we used a GFR cutoff of 100 mL/min/1.73 m^2^ to identify patients with possible ARC (pARC). Using the MDRD calculation, 83 patients (16.8%) had pARC. By CKD-EPI calculations, 82 patients (16.6%) had pARC. Table 2 provides a detailed breakdown of GFR for the population as calculated by MDRD and CKD-EPI equations.

**Table 1:** Patient characteristics by GFR.

Characteristics	All patients without ESRD (494)	Patients with GFR <130 (467)	Patients with GFR >130^a^ (27)	P value^b^
Age, yrs	59.9 ± 15.8	61.0 ± 15.0	40.5 ± 16.2	<0.001
Male, % (#)	52.6 (260)	53.1 (248)	44.4 (12)	0.381
African-American, % (n)	30.0 (148)	27.8 (130)	66.7 (18)	<0.001
Mechanical ventilation, % (n)	19.2 (95)	19.7 (92)	11.1 (3)	0.271
Bone marrow transplant, % (n)	4.7 (23)	4.3 (20)	11.1 (3)	0.124
Solid organ transplant, % (n)	3.8 (19)	4.1 (19)	0	0.615
CHF, % (n)	14.0 (69)	14.8 (69)	0	0.022
COPD, % (n)	14.2 (70)	14.6 (68)	7.4 (2)	0.403
Diabetes mellitus, type 2, % (n)	28.5 (141)	29.3 (137)	14.8 (4)	0.104
Solid organ malignancy, % (n)	27.9 (138)	28.7 (134)	14.8 (4)	0.118
Leukemia, % (n)	19.2 (95)	18.4 (86)	33.3 (9)	0.056
Lymphoma, % (n)	6.1 (30)	6.2 (29)	3.7 (1)	1
Cirrhosis, % (n)	5.5 (27)	5.8 (27)	0	0.387
Antibiotics within 30 days, % (n)	38.3 (189)	37.5 (175)	51.9 (14)	0.135
Healthcare exposure, % (n)	69.2 (342)	68.7 (321)	77.8 (21)	0.322
MDR, % (n)	18.6 (92)	18.8 (88)	14.8 (4)	0.8
Time to appropriate antibiotics (hours)	2.4 [1.1–5.0]	2.4 [1.1–5]	2.1 [1.0–5.3]	0.930
Immunosuppressed, % (n)	36.8 (182)	36.6 (171)	40.7 (11)	0.666
Charlson Comorbidity Score	1.7 ± 1.3	1.7 ± 1.3	0.48 ± 0.7	<0.001
APACHE II score	13.3 ± 5.3	13.4 ± 5.3	10.2 ± 5.3	0.002
Patient origin, % (n)				0.845
Nursing home, SNF, or LTACH	8.5 (42)	8.6 (40)	7.4 (2)	
Community	54.7 (270)	54.8 (256)	51.9 (14)	
OSH	10.3 (51)	10.5 (49)	7.4 (2)	
In hospital	26.5 (131)	26.1 (122)	33.3 (9)	
Infection source, % (n)				
Central venous catheter	8.9 (44)	8.8 (41)	11.1 (3)	0.724
Genitourinary	42.5 (210)	42.8 (200)	37.0 (10)	0.554
Pulmonary	5.5 (27)	5.1 (24)	11.1 (3)	0.177
Gastrointestinal	16.4 (81)	17.1 (80)	3.7 (1)	0.103
Unknown	24.5 (121)	24.0 (112)	33.3 (9)	0.272
Other^c^	2.2 (11)	2.1 (10)	3.7 (1)	0.465
Sepsis	34.4 (170)	33.2 (155)	55.6 (15)	0.051 for all categories
Severe sepsis	37.7 (186)	38.1 (178)	29.6 (8)	
Septic shock	27.9 (138)	28.7 (134)	14.8 (4)	
Elevated lactate (>2.1)	2.4 [1.4–4.2]	2.4 [1.4–4.2]	2.0 [1.3–3.9]	0.629
LOS	7.7 [4.7–19.6]	7.7 [4.6–19.6]	8.8 [4.8–20.8]	0.644
ICU LOS	0.8 [0–4.8]	0.9 [0–4.7]	0 [0–7.6]	0.913
Thirty-day mortality	13.0 (64)	13.1 (61)	11.1 (3)	1

^a^GFR >130 by MDRD or CKD-EPI.

^b^p value for comparison of patients with ARC to those without ARC.

^c^Types of infections in the other category include central nervous system, skin and soft tissue, vascular graft, muscle, joint, osteomyelitis, and gynecologic. ARC: augmented renal clearance. CHF: congestive heart failure; COPD: chronic obstructive pulmonary disease; CKD: chronic kidney disease; ESRD: end-stage renal disease; MDR: multi-drug resistance; APACHE II: Acute physiology and chronic health evaluation II; SNF: skilled nursing facility; LTACH: long-term acute care hospital; OSH: outside hospital; CNS: central nervous system.

**Table 2:** Proportions of patients falling into various GFR ranges as assessed by MDRD and CKD-EPI equations.

	MDRD n (%)	CKD-EPI n (%)
GFR ≥130 mL/min/1.73 m^2^	25 (5.1%)	15 (3.0%)
130>GFR≥90 mL/min/1.73 m^2^	92 (18.6%)	118 (23.9%)
90>GFR≥60 mL/min/1.73 m^2^	123 (24.9%)	118 (23.9%)
60>GFR≥30 mL/min/1.73 m^2^	164 (33.2%)	154 (31.2%)
30>GFR≥15 mL/min/1.73 m^2^	68 (13.8%)	65 (13.2%)
GFR <15 mL/min/1.73 m^2^	22 (4.5%)	24 (4.9%)

MDRD: Modification of Diet in Renal Disease. CKD-EPI: Chronic Kidney Disease Epidemiology Collaboration. GFR: glomerular filtration rate

Seventy-three patients received two antibiotics with Gram-negative activity in the 12 hours after positive culture and 4 received 3 antibiotics with Gram-negative activity in the 12 hours after positive culture. The predominant Gram-negative antibiotic received was cefepime with 52.2% (n = 266) of patients receiving it within 12 hours, followed by piperacillin-tazobactam at 20% (n = 102), meropenem at 15.7% (n = 80), and ceftriaxone at 12.0% (n = 61)–see Table 3. Patients with ARC by either MDRD or CKD-EPI calculations (n = 27) received predominantly cefepime at 63.0% (n = 17), 14.8% (n = 4 each) received meropenem or ceftriaxone, and 11.1% (n = 3) received piperacillin-tazobactam (Table 3). Ampicillin-sulbactam, ciprofloxacin, ertapenem, and aztreonam were also administered to one patient each. Three patients with ARC received 2 drugs and 1 patient received 3 drugs within 12 hours of positive blood cultures.

**Table 3:** Proportions of patients receiving different antibiotics within 12 hours of positive blood culture.

	All patients(510), % (n)	Patients without ARC (467), % (n)	Patients with ARC by MDRD or CKD-EPI (27), % (n)
Cefepime	52.2 (266)	51.2 (239)	63.0 (17)
Piperacillin-tazobactam	20.0 (102)	20.6 (96)	11.1 (3)
Meropenem	15.7 (80)	15.4 (72)	14.8 (4)
Ceftriaxone	12.0 (61)	12.0 (56)	14.8 (4)
Other	14.7 (75)	14.8 (4)	15.2 (71)

No differences between ARC and non-ARC patients were significant. Other = ampicillin, ampicillin-sulbactam, aztreonam, cefotetan, ceftaroline, ertapenem, ciprofloxacin, doxycycline, levofloxacin, trimethoprim-sulfamethoxazole. ARC: augmented renal clearance. MDRD: Modification of Diet in Renal Disease. CKD-EPI: Chronic Kidney Disease Epidemiology Collaboration.

From our previous multivariate logistic regression analysis, sepsis severity, African-American race, cirrhosis, solid organ malignancy, transfer from an OSH, and APACHE-II score were risk factors for mortality [15]. Addition of augmented renal clearance to the model did not significantly change the model and ARC was not statistically associated with mortality (S1 Table). A GFR greater than 100 mL/min/1.73 m^2^ was also not associated with mortality. Excluding patients that were discharged on hospice (n = 10, 1 with ARC) did not significantly affect the logistic regression models for mortality or ARC.

For a sensitivity analysis, we excluded patients that received ceftriaxone as renal clearance should not alter therapeutic efficacy. There were 61 patients that received ceftriaxone, leaving 433 available for analysis, 23 with ARC. Similarly to the entire cohort, addition of augmented renal clearance to the model did not significantly change the model and ARC was not statistically associated with mortality (p = 0.36).

After multivariate logistic regression analysis, age, African-American race, and sepsis severity were found to be predictors of the presence of ARC (Table 4). The likelihood of having ARC increased 6.4% with each decrease in year of age. Being African-American had an odds ratio for the presence of ARC of 3.45 with 95% confidence intervals of 1.40–8.50. An increase in sepsis severity was associated with a 36% lower likelihood of having ARC.

**Table 4:** Risk factors associated with ARC, as determined by multivariate logistic regression.

Factor	Odds ratio [95% confidence interval]
Age	0.93 [0.91–0.96]
African-American race	3.45 [1.40–8.50]
Sepsis severity	0.54 [0.30–0.97]

African-American race and younger age significantly increased the risk for having ARC, while increasing severity of sepsis was associated with a lower incidence of ARC.

## Discussion

We found that ARC was not a predictor of mortality among patients with *Enterobacteriaceae* bloodstream infections receiving appropriate initial antimicrobial therapy within 12 hours of positive blood cultures being drawn. Predictors of mortality in the cohort were African-American race, transfer from an OSH, increasing APACHE-II scores, underlying malignancy, and cirrhosis, which are known risk factors for mortality in sepsis and reflect acute and chronic illness severity [15]. Interestingly, the percentage of patients with ARC was <5%, much lower than reported rates in the literature [1–5, 7, 10, 20, 21]. However, ARC varies by patient population, being more common in trauma and younger patients, which were not the predominant demographics of our cohort. In addition, we assessed ARC retrospectively without measured urinary creatinine clearance, which is known to underestimate the prevalence of ARC by up to 30% [5–9].

We found that African-American race, younger age, and lower sepsis severity were associated with a higher likelihood of ARC. Younger age and lower sepsis severity have previously been shown to increase the incidence of ARC [1–4]. Although African-American race is known to effect estimates of GFR, African-American race has not previously been reported to increase the rate of ARC. Leukemia was more common in patients with ARC, but this was confounded by younger age in the ARC group with leukemia as compared to those with leukemia and no ARC.

Our study is limited in several ways. The retrospective nature of the study makes it difficult to elucidate possible confounders that could have biased the outcome measures. This was a single-center study and results may not be generalizable to other centers. Augmented renal clearance is difficult to definitively establish using GFR calculators rather than prospectively collected urinary creatinine clearance measurements. In addition, beta-lactam levels are not available at our institution, which could alter the effect of ARC on mortality if levels were subtherapeutic in either the ARC or non-ARC group. The use of disc diffusion methods to determine antimicrobial susceptibility is a potential limitation, as we do not have minimum inhibitory concentrations (MIC) available to correlate with outcomes. However, previous studies have shown good correlation between broth microdilution (BMD) and disc diffusion [22]. In some cases, BMD techniques may misclassify resistant isolates as susceptible [23], which would bias results towards the null. It is unknown whether misclassification of resistant isolates as susceptible with BMD or undetected MIC differences when using disc diffusion methodology would have a greater impact on our study conclusions. As each susceptibility testing method has its limitations, our study provides helpful insight regarding ARC and outcomes in the context of disc diffusion susceptibility testing. We did not study outcomes in patients with Gram-positive infections or non-*Enterobacteriaceae* Gram-negative infections. It is possible that ARC would be more important in determining clinical outcomes in populations treated with different antibiotics for other types of pathogens and this is an area ripe for future studies. In conclusion, ARC was not a predictor of mortality in patients that all received appropriate initial antimicrobial therapy, though the incidence of ARC in our cohort was low. We found that African-American race, age, and sepsis severity were important predictors of the presence of ARC. Our results provide impetus for further prospective data collection on ARC in medical ICU populations. Future studies can assess similar outcomes in patients with Gram-positive or Gram-negative non-*Enterobacteriaceae* sepsis, and determine whether ARC is an important outcome predictor among patients receiving appropriate initial antimicrobial therapy in those groups.

## Supporting information

S1 Data SPSS file containing data relevant for this study. (SAV)

S1 Table Risk factors associated with 30-day all-cause mortality, as determined by multivariate logistic regression. APACHE II: Acute physiology and chronic health evaluation II; OSH: outside hospital.
(DOCX)

## References

[1] Claus BO, Hoste EA, Colpaert K, Robays H, Decruyenaere J, De Waele JJ. Augmented renal clearance is a common finding with worse clinical outcome in critically ill patients receiving antimicrobial therapy. J Crit Care. 2013;28: 695–700. doi: 10.1016/j.jcrc.2013.03.003 23683557 10.1016/j.jcrc.2013.03.003

[2] Udy AA, Roberts JA, Shorr AF, Boots RJ, Lipman J. Augmented renal clearance in septic and traumatized patients with normal plasma creatinine concentrations: identifying at-risk patients. Crit Care. 2013;17: R35 PMCID: PMCPMC4056783. doi: 10.1186/cc12544 23448570 10.1186/cc12544 PMC4056783

[3] De Waele JJ, Dumoulin A, Janssen A, Hoste EA. Epidemiology of augmented renal clearance in mixed ICU patients. Minerva Anestesiol. 2015;81: 1079–1085. 25697881

[4] Kawano Y, Morimoto S, Izutani Y, Muranishi K, Kaneyama H, Hoshino K, et al Augmented renal clearance in Japanese intensive care unit patients: a prospective study. J Intensive Care. 2016;4: 62 doi: 10.1186/s40560-016-0187-7 27729984 10.1186/s40560-016-0187-7 PMC5048448

[5] Baptista JP, Neves M, Rodrigues L, Teixeira L, Pinho J, Pimentel J. Accuracy of the estimation of glomerular filtration rate within a population of critically ill patients. J Nephrol. 2014;27: 403–410. doi: 10.1007/s40620-013-0036-x 24446348 10.1007/s40620-013-0036-x

[6] Adnan S, Ratnam S, Kumar S, Paterson D, Lipman J, Roberts J, et al Select critically ill patients at risk of augmented renal clearance: experience in a Malaysian intensive care unit. Anaesth Intensive Care. 2014;42: 715–722. 25342403 10.1177/0310057X1404200606

[7] Barletta JF, Mangram AJ, Byrne M, Hollingworth AK, Sucher JF, Ali-Osman FR, et al The importance of empiric antibiotic dosing in critically ill trauma patients: Are we under-dosing based on augmented renal clearance and inaccurate renal clearance estimates? J Trauma Acute Care Surg. 2016;81: 1115–1121. doi: 10.1097/TA.0000000000001211 27533906 10.1097/TA.0000000000001211

[8] Udy AA, Morton FJ, Nguyen-Pham S, Jarrett P, Lassig-Smith M, Stuart J, et al A comparison of CKD-EPI estimated glomerular filtration rate and measured creatinine clearance in recently admitted critically ill patients with normal plasma creatinine concentrations. BMC Nephrol. 2013;14: 250 doi: 10.1186/1471-2369-14-250 24225349 10.1186/1471-2369-14-250 PMC3840599

[9] Bragadottir G, Redfors B, Ricksten SE. Assessing glomerular filtration rate (GFR) in critically ill patients with acute kidney injury—true GFR versus urinary creatinine clearance and estimating equations. Criti Care. 2013;17: R108. 10.1186/cc12777 PMC4056314 23767877

[10] Huttner A, Von Dach E, Renzoni A, Huttner BD, Affaticati M, Pagani L, et al Augmented renal clearance, low beta-lactam concentrations and clinical outcomes in the critically ill: an observational prospective cohort study. Int J Antimicrob Agents. 2015;45: 385–392. doi: 10.1016/j.ijantimicag.2014.12.017 25656151 10.1016/j.ijantimicag.2014.12.017

[11] Hirai K, Ishii H, Shimoshikiryo T, Shimomura T, Tsuji D, Inoue K, et al Augmented renal clearance in patients With febrile neutropenia is associated with increased risk for subtherapeutic concentrations of vancomycin. Ther Drug Monit. 2016;38: 706–710. doi: 10.1097/FTD.0000000000000346 27681114 10.1097/FTD.0000000000000346

[12] Baptista JP, Sousa E, Martins PJ, Pimentel JM. Augmented renal clearance in septic patients and implications for vancomycin optimisation. Int J Antimicrob Agents. 2012;39: 420–423. doi: 10.1016/j.ijantimicag.2011.12.011 22386742 10.1016/j.ijantimicag.2011.12.011

[13] Udy AA, Varghese JM, Altukroni M, Briscoe S, McWhinney BC, Ungerer JP, et al Subtherapeutic initial beta-lactam concentrations in select critically ill patients: association between augmented renal clearance and low trough drug concentrations. Chest. 2012;142: 30–39. doi: 10.1378/chest.11-1671 22194591 10.1378/chest.11-1671

[14] Roberts JA, Paul SK, Akova M, Bassetti M, De Waele JJ, Dimopoulos G, et al DALI: defining antibiotic levels in intensive care unit patients: are current beta-lactam antibiotic doses sufficient for critically ill patients? Clin Infect Dis. 2014;58: 1072–1083. doi: 10.1093/cid/ciu027 24429437 10.1093/cid/ciu027

[15] Burnham JP, Lane MA, Kollef MH. Impact of sepsis classification and multidrug-eesistance status on outcome among patients treated with appropriate therapy. Crit Care Med. 2015;43: 1580–1586. doi: 10.1097/CCM.0000000000001013 25855900 10.1097/CCM.0000000000001013 PMC4672994

[16] Knaus WA, Draper EA, Wagner DP, Zimmerman JE. APACHE II: a severity of disease classification system. Crit Care Med. 1985;13:818–829. 3928249

[17] Levey AS, Stevens LA, Schmid CH, Zhang YL, Castro AF 3rd, Feldman HI, et al A new equation to estimate glomerular filtration rate. Ann Intern Med. 2009;150: 604–612. 19414839 10.7326/0003-4819-150-9-200905050-00006 PMC2763564

[18] Levey AS, Coresh J, Greene T, Stevens LA, Zhang YL, Hendriksen S, et al Using standardized serum creatinine values in the modification of diet in renal disease study equation for estimating glomerular filtration rate. Ann Intern Med. 2006;145: 247–254. 16908915 10.7326/0003-4819-145-4-200608150-00004

[19] CLSI. Performance Standards for Antimicrobial Susceptibility Testing; Twenty-Fifth Informational Supplement. CLSI document M100-S25. Wayne, PA: Clinical and Laboratory Standards Institute; 2015.

[20] Udy AA, Baptista JP, Lim NL, Joynt GM, Jarrett P, Wockner L, et al Augmented renal clearance in the ICU: results of a multicenter observational study of renal function in critically ill patients with normal plasma creatinine concentrations. Crit Care Med. 2014;42: 520–527. doi: 10.1097/CCM.0000000000000029 24201175 10.1097/CCM.0000000000000029

[21] May CC, Arora S, Parli SE, Fraser JF, Bastin MT, Cook AM. Augmented renal clearance in patients with subarachnoid hemorrhage. Neurocrit Care. 2015;23: 374–379. doi: 10.1007/s12028-015-0127-8 25761425 10.1007/s12028-015-0127-8 PMC12968823

[22] Jones RN, Craig WA, Ambrose PG, Dudley MN, Pottumarthy S. Reevaluation of Enterobacteriaceae MIC/disk diffusion zone diameter regression scattergrams for 9 beta-lactams: adjustments of breakpoints for strains producing extended spectrum beta-lactamases. Diagn Microbiol Infect Dis. 2005;52:235–346. doi: 10.1016/j.diagmicrobio.2005.02.006 16105568 10.1016/j.diagmicrobio.2005.02.006

[23] Luc M. A Comparison of Disc Diffusion and Microbroth Dilution Methods for the Detection of Antibiotic Resistant Subpopulations in Gram Negative Bacilli. University of Washington: University of Washington; 2015.

