# Supplementary material for: Augmented renal clearance is not a risk factor for mortality in Enterobacteriaceae bloodstream infections treated with appropriate empiric antimicrobials
Source: PLoS One. 2017 Jul 5;12(7):e0180247. doi: 10.1371/journal.pone.0180247 (PMC5497982; doi:10.1371/journal.pone.0180247)
Supplement: S1 Table — APACHE II: Acute physiology and chronic health evaluation II; OSH: outside hospital. (DOCX) [file pone.0180247.s002.docx]

**S1 Table**

| **Factor** | **Odds ratio [95% confidence interval]** |
| --- | --- |
| Sepsis severity | 1.96 [1.20-3.20] |
| African-American race | 3.27 [1.68-6.35] |
| APACHE II (1-point increments) | 1.15 [1.08-1.22] |
| Solid organ cancer | 3.48 [1.83-6.61] |
| Cirrhosis | 7.52 [2.73-20.7] |
| Patient origin from OSH | 3.34 [1.43-7.80] |
| Augmented renal clearance | 0.75 [0.24-2.31] |

APACHE II: Acute physiology and chronic health evaluation II; OSH: outside hospital.
